# Detrusor-Overactivity-Related Voiding in Women Mimics Bladder Outflow Obstruction and Conceals Underactivity

**DOI:** 10.5152/tud.2023.22213

**Published:** 2023-07-01

**Authors:** Takeya Kitta, Shinya Kobayashi, Mio Togo, Hiroki Chiba, Madoka Higuchi, Naohisa Kusakabe, Mayuko Tsukiyama, Mifuka Ouchi, Yui Abe-Takahashi, Nobuo Shinohara

**Affiliations:** 1 Department of Renal and Urologic Surgery, Asahikawa Medical University, Asahikawa, Japan; 2 Miyanosawa Nephro–Urology Clinic, Sapporo, Hokkaido, Japan; 3 Department of Renal and Genitourinary Surgery, Graduate School of Medicine, Hokkaido University, Sapporo, Hokkaido, Japan

**Keywords:** Overactive detrusor, neurogenic bladder, bladder outflow obstruction, detrusor underactivity, urodynamics

## Abstract

**Objective:**

Urodynamics of the storage phase showing detrusor overactivity is common in neurogenic bladder patients. Terminal detrusor overactivity, which is defined by involuntary detrusor contraction that cannot be inhibited, causes urinary incontinence. Such incontinence causes a unique voiding in neurogenic bladder patients. During the voiding phase, the detrusor pressure at Qmax (Pdet.Qmax)/maximum flow rate (Qmax) (P/Q) is the gold standard for differentiating between detrusor underactivity and bladder outflow obstruction. We investigated whether a valid identification of lower urinary tract dysfunction could be established from P/Q assessment of detrusor overactivity-related voiding patients.

**Methods:**

This study evaluated 2 types of voiding. Detrusor overactivity-related voiding is involuntary detrusor contraction that results in micturition or voiding after permission to void when detrusor overactivity has occurred, while voluntary voiding is voiding voluntarily after permission to void and without terminal detrusor overactivity. We evaluated female patients with neurogenic bladder who could undergo micturition without catheterization. A pressure flow study compared the 2 groups.

**Results:**

Comparison of the detrusor overactivity-related voiding group (n = 20) and the voluntary voiding group (n = 12) found statistically significant differences with a lower Qmax and higher Pdet.Qmax (*P* = .01) in the detrusor overactivity-related voiding group. The linear regression analysis P/Q plot showed the positivity and negativity value of the slope that was reversed in the 2 groups (−0.089 vs. 0.198).

**Conclusion:**

Current results showed different P/Q plot patterns between 2 types of voiding in patients with neurogenic bladder. These findings suggest there is increased detrusor pressure observed in detrusor overactivity-related voiding that mimics outflow obstruction.

**Main Points**

- Urodynamics of the storage phase showing detrusor overactivity is frequent in neurogenic bladder patients.
- The purpose of this study was to see if detrusor pressure and flow rate evaluation of DO-related voiding could be used to make a good diagnosis of lower urinary tract dysfunction.
- Detrusor overactivity-related voiding mimics outflow obstruction and conceals underactivity.

## Introduction

Voiding dysfunctions can be induced by increasing lower urinary tract (bladder neck or urethral) resistance, impairing bladder contractility, or both. Pressure flow studies are the gold standard for diagnosing bladder outflow obstruction (BOO) and other lower urinary tract dysfunctions. When using urodynamics to examine the storage phase, detrusor overactivity (DO) is common during urodynamic testing in patients with overactive bladder (OAB) and is characterized by the occurrence of non-prevented bladder contractions during the filling phase. Moreover, there are 2 frequent patterns shown by DO, which include phasic DO and terminal DO.^1^ Phasic DO is characterized by involuntary detrusor contractions, which may or may not be voluntarily inhibited. Terminal DO is characterized by a single involuntary detrusor contraction that cannot be inhibited, thus, causing urinary incontinence. Detrusor underactivity (DU) according to the International Continence Society (ICS) definition is “the presence of low detrusor pressure or short detrusor contraction time, usually in combination with a low urine flow rate that results in prolonged bladder emptying and/or failure to achieve complete bladder emptying within a normal time span.”^1^ However, there has been no report that has specifically evaluated the validity of the pressure flow study parameters recorded during voiding subsequent to terminal DO.

Our current study focused on female patients in order to avoid the complexity of the male composition of DU and BOO. The purpose of this research is a valid diagnosis of lower urinary tract dysfunction that can be established based on a pressure flow study analysis of DO-related voiding patients with a neurogenic bladder.

## Material and Methods

This study was performed in accordance with the Declaration of Helsinki and approved by the Scientific Ethics Committee of Hokkaido University Hospital. This study was conducted in a retrospective manner, and all data were retrospectively investigated based on the patient’s electronic medical charts done with Institutional Review Board permission. This is not a forward-looking clinical trial, so informed consent from all patients was not required. (We applied an opt-out method to obtain consent for this retrospective study). Verbal informed consent was obtained from patients who agreed to take part in the study.

Female patients with neurogenic bladder who were able to undergo micturition without any catheterization were included. Multichannel urodynamic evaluations were performed on all patients in accordance with the report of the Good Urodynamic Practice Guidelines.^2^ All patients were confirmed to be free of significant pelvic organ prolapse when urodynamic testing was performed. We used routinely 2-lumen transurethral catheters. The 6Fr catheter was used to infuse room temperature saline at 50 mL/min and for assessing bladder pressure. Intraabdominal pressure was assessed using a 9Fr rectal catheter. The detrusor pressure was calculated by subtracting the intraabdominal pressure from the bladder pressure. Detrusor overactivity was characterized as evidence of voluntary detrusor contractions occurring while bladder filling (phasic detrusor overactivity) or an uncontrolled detrusor contraction occurs at bladder cystometric capacity that generally resulted in voiding (terminal DO).^3^ Detrusor overactivity was defined in the current study as an involuntary rise of detrusor pressure greater than 5 cm H_2_O during the filling phase. Exclusion criteria included pelvic organ prolapse, complete spinal cord injury that prevented micturition, transurethral surgery, bladder cancer, and urinary tract calculi.

**Figure 1.**
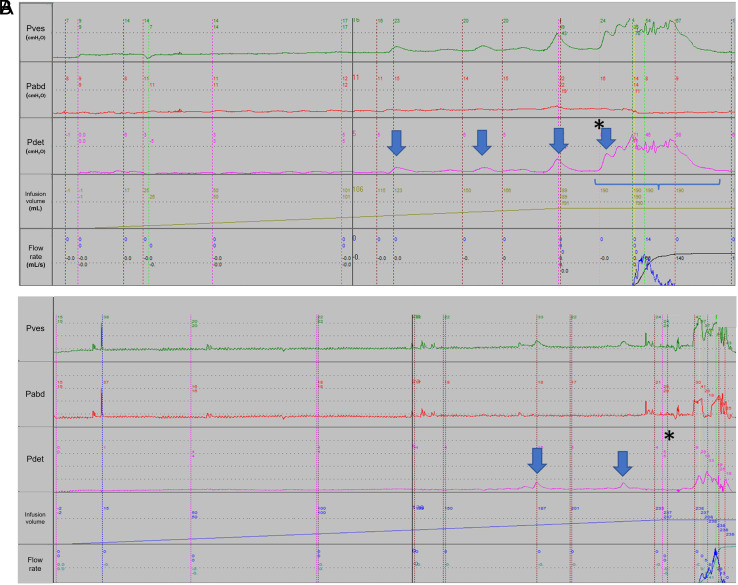
Detrusor overactivity-related voiding (A); involuntary detrusor contraction accompanied by voiding was observed. Voluntary voiding (B); voiding voluntarily after permission to void and without terminal detrusor overactivity. Arrows indicate places in the Pdet (detrusor pressure) that show detrusor overactivity. Asterisks indicate “permission to void.” The blue bracketed area represents the detrusor-overactivity -related voiding.

Each subject underwent pressure flow studies that evaluated maximum flow rate (Qmax) (mL/s) and detrusor pressure at Qmax (Pdet.Qmax) (cm H_2_O). Two types of voiding were defined in this study. The first involved DO-related voiding micturition or voiding after permission to void under DO occurred (Figure 1A). In other words, DO-related voiding can be described as a group of patients who can maintain self-restraint to some extent even if DO occurs during the urine storage period and who are able to urinate voluntarily if urinated in this state (DO → urination permitted → urination). The second involved voluntary voiding in which voiding occurred voluntarily after permission to void and without terminal DO (Figure 1B). This study specifically focused on the parameters of the voiding phase in order to explore the difference between the 2 types of voiding.

### Statistical Analysis

Statistical analyses were conducted using GraphPad Prism for Windows Ver. 6.01 (GraphPad Software, San Diego, Calif, USA). A Student’s paired* t*-test was used to do a statistical analysis of parametric results. The linear regression analysis was carried out and compared using Qmax and Pdet.Qmax. All statistical results were considered significant at a *P*-value of less than .05.

## Results

**Figure 2.**
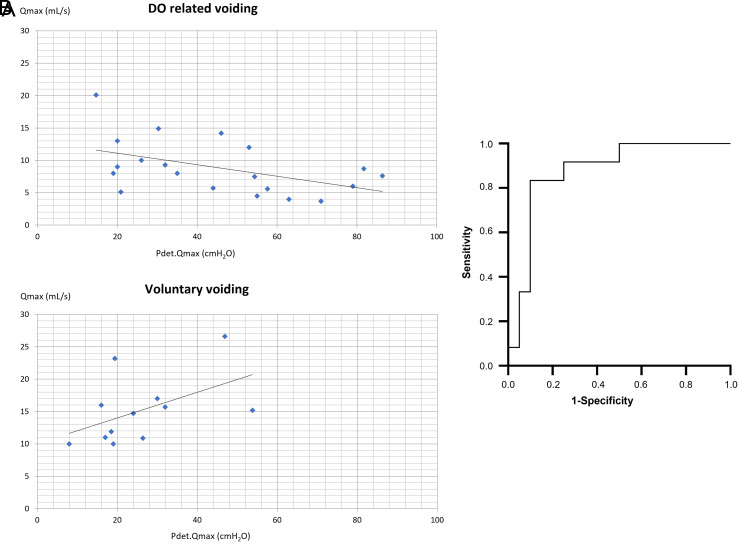
A linear regression analysis (P/Q) (Pdet.Qmax Qmax) plot shows that the value of the slope was reversed in positivity and negativity. The detrusor overactivity-related voiding *y*-intercept is 12.90 and the slope is −0.089, while for the voluntary voiding, the *y*-intercept is 10.06 and the slope is 0.198 (A). We performed ROC curves. BOOIf (Pdet.Qmax − 2.2 × Qmax) was calculated from the parameters Qmax and Pdet.Qmax (which are linked to each other) when performing ROC. The ROC analysis showed significant differences in the BOOIf compared to the non-discrimination line. Furthermore, the area under the ROC curve was 0.875 (B).

The study evaluated 32 women (mean age 55.1 ± 20.5 years, range 16-85 years) with lower urinary tract symptoms. Table 1 lists the causes of the neurogenic bladder. There were no significant differences in age between the 2 groups (60.2 vs. 46.6). When the DO-related voiding group was compared to the voluntary voiding group, the following statistically significant differences that were observed included finding that the Qmax was lower and the Pdet.Qmax was higher (*P* = .01) in the DO-related voiding group (Table 2). Moreover, for the linear regression analysis (P/Q (Pdet.Qmax Qmax) plot), the value of the slope was reversed for positivity and negativity, with a DO-related voiding *y*-intercept of 12.90 and a slope of −0.089, while for the voluntary voiding, the *y*-intercept was 10.06 and the slope was 0.198 (Figure 2A). And to evaluate the usefulness of P/Q (Qmax and Pdet.Qmax) plots for identifying patients where parameters mimic outflow obstruction and conceal underactivity, we performed ROC curves. BOOIf (Pdet.Qmax − 2.2 × Qmax) was calculated from the parameters Qmax and Pdet.Qmax, when performing ROC. The ROC analysis showed significant differences in the BOOIf compared to the non-discrimination line, and the area under the ROC curve was 0.875 (Figure 2B).

## Discussion

During the voiding phase, Pdet.Qmax/Qmax (P/Q) is used to simultaneously measure the Pdet and flow rate. P/Q assessment is considered to be the gold standard for quantifying and grading BOO and for differentiating between BOO and DU. This study is the first to demonstrate different patterns in the P/Q plot between the 2 voiding patterns in neurogenic bladder patients. Although all of the patients evaluated did not appear to have BOO anatomically, the P/Q plot showed that patients with DO-related voiding had a significantly high pressure/low flow pattern, which might be mimicking outflow obstruction. Our current study defines DO-related voiding, which include not only a voiding following terminal DO but also DO with permission to void in conjunction with the occurrence of DO. In case of a voiding following terminal DO, the patient was encouraged to continue voiding as long as possible. Furthermore, it has been confirmed that all the patients with DO-related voiding mainly urinate in this manner on a daily basis. The purpose of our current study was to clarify if DO-related voiding affects the P/Q pattern. To my knowledge, there have been few previous reports that have focused on DO and micturition. Even though Valentini et al^4^ evaluated the urodynamic characteristics of the 2 patterns of DO, they included all patients with DO during their urodynamic evaluations and did not clarify the DO during voiding.

Bladder outflow obstruction is identified as a high pressure that achieves only a low flow rate.^1^ The presence of benign prostatic hyperplasia often brings bias when trying to understand the pathophysiology of micturition or when specifically evaluating BOO as a neurogenic cause. Therefore, our study focused on female patients without benign prostatic hyperplasia in order to minimize the prostate bias. Bladder outflow obstruction in women patients has traditionally been hard to identify because the anatomy and physiology of micturition differ from that in men. The normal pressure generated within the bladder by the detrusor muscles is significantly lower in women as compared to men. Our current study compared patients with DO-related voiding who appeared to have a BOO pattern with voluntary voiding patients (lower Qmax and higher Pdet.Qmax), even though yet we do not have a validated female nomogram. As a result, there is currently a lack of established diagnostic criteria or nomograms, and the prevalence of BOO in women is likely to be underestimated.^5^ Blaivas and Groutz developed a nomogram from urodynamic data from women who have BOO at their institution.^5^ This nomogram has proved to be a valuable tool in aiding with the proper identification of BOO in women. Furthermore, the BOO index of Solomon–Greenwell has also been developed.^6^ This index is calculated using the formula “BOOIf = Pdet.Qmax − 2.2 × Qmax,” a BOOIf over 18 indicates that obstruction is almost certain (over 90%). Although their report excluded patients with overt neurological abnormalities, there is no nomogram that can be adapted for our considerations. When we apply the results of our current data, the Solomon–Greenwell nomogram diagnosed BOO in 10 patients within those with DO-related voiding (50%). In contrast, only 1 patient (8.3%) was diagnosed as a BOO patient with voluntary voiding. Moreover, the BOOIf of the DO-related voiding was significantly higher as compared to the voluntary voiding in this study (26.0 ± 6.4 vs. −7.5 ± 3.6, *P* < .01). We performed ROC curve (Figure 2B) using BOOIf. In the present study, it was confirmed that the anatomically unobstructed patient group had higher obstruction (BOOIf) in patients with DO-related voiding. In other words, it was confirmed that attention is required when conducting PFS tests in patients who are DO urinating. It may become a relevant parameter for the diagnostic criteria of BOO in women if the number of cases increases in the future.

As previously discussed, voiding dysfunction in women could potentially be related to DU and/or BOO. A contraction of diminished power and/or length that leads to protracted bladder emptying and/or inability to accomplish full bladder emptying within a typical time range is classified as DU. Unfortunately, there are no well-defined urodynamic parameter thresholds for DU’s low-pressure low-flow pattern.^7^ In our current study, bladder contractility (which was evaluated using the projected isovolumetric pressure (PIP1) (Pdet.Qmax + Qmax)) was not significantly different for the 2 types of voiding (54.3 ± 20.6 vs. 41.1 ± 15.8, *P* = .07). This could be due to the fact that outflow obstruction conceals DU in DO-related voiding. Furthermore, when under a DO-related voiding condition, it can be difficult to detect DU from the urodynamics parameters.

Today, urodynamic studies have been an essential instrument for assessing symptoms of the lower urinary tract. In addition, electromyography (EMG) has also an important tool for diagnosis. However, especially “surface” EMG may contain artifacts, which can appear to be increased activity during voiding. Moreover, needle EMG is invasive and uncomfortable. Therefore, if an accurate diagnosis can be made by other methods, this would mean that an EMG would not be necessary during a pressure flow study. Voluntary voiding is prompted by the release of tonic inhibition from the supra-pontine centers. As a result, this allows the pontine micturition center (PMC) to trigger the micturition reflex. This procedure is mediated by the relaxation of the external striated urethral sphincter, pelvic floor muscles, and internal smooth sphincter, starting with the contraction of the detrusor muscle. However, inappropriate sphincter activity during voiding detrusor sphincter dyssynergia (DSD) causes functional BOO and inhibits the micturition reflex. This condition is most common after supra-sacral spinal cord injury and usually involves the external sphincter (external-DSD). Pseudo-DSD has been defined as dysfunctional voiding patients who can be BOO.^8,9^ The diagnosis of dysfunctional voiding is described as an enhanced external sphincter activity during voiding in neurologically normal women.^10^ In our current study, we focused on voiding with DO (DO-related voiding) patients with neurogenic bladder. Our results showed that while sphincter dysfunction during DO-related voiding was similar, it may not be DSD. Furthermore, these patients were also not able to relax their sphincters properly after being permitted to void. In actuality, when DO starts, reflex contraction of the pelvic floor muscle is a biological reaction. However, if the patient is able to avoid urinary incontinence by continuing to voluntarily contract the pelvic floor muscles, the DO eventually ceases. It has been reported that women with long-standing DO seem to have increased urethral closure pressures,^11^ which suggests that they have effectively done resistance training in order to strengthen their sphincter, and thus, reduced their risk of DO incontinence.

Advances in functional brain imaging have elucidated the location of anatomical nuclei within the brain that are active in relation to the micturition reflex.^12^ While we are resisting micturition, afferent impulses climb to synapse in the periaqueductal gray of the midbrain. If these exceed a certain threshold, the micturition reflex is triggered, and a signal is sent to the PMC. Pontine micturition center excitation can cause urinary sphincter and pelvic floor muscles relaxation and an excitatory parasympathetic pathway that leads to detrusor contraction. Although the primary mechanism of OAB has not yet been elucidated, there are many reports that have examined the brain mechanisms during OAB.^13^ The cerebellum and parietal lobe are activated during withholding micturition and the pelvic floor muscles contraction.^14^ Kitta et al reported that patients with Parkinson’s disease exhibited significant brain activation in many brain regions including the PAG during DO. However, the PMC was not activated during DO.^15^ Based on these reports, it appears that the DO supra-pontine centers did not allow the PMC to trigger the voiding reflex. Thus, this neural control may induce (pseudo-)DSD, even when patients were given permission to void (allowed).

There were several limitations to our current study. First, this study is a retrospective study, the prevalence of neurogenic bladder present in clinical practice may differ. And the number of this study was small, which could have affected the robustness of our results. Nonetheless, the findings of this study indicate the significance of important information for future treatments of patients with neurogenic bladder. Second, there is currently no established female BOO definition. Because of this, we chose to use the Solomon–Greenwell definition, as it has recently become widely used. Third, the exact cause of neurogenic bladder in some patients was unknown. Neurogenic bladder normally refers to lower urinary tract problems due to injury or dysfunction of the central/peripheral nervous system involved in the micturition control. In our study, we included female patients who had voiding dysfunction including DO during urodynamic evaluations. As a fourth limitation, it was difficult to rule out the absolute absence of anatomical lower urinary tract obstruction. Although additional studies will need to be undertaken to define DO-related voiding in women, our results add new information to the presently accessible information database.

In conclusion, findings for the current study suggest that increased detrusor pressure was observed during voiding in the DO-related voiding group subsequent to terminal DO and DO mimics outflow obstruction in neurogenic detrusor overactivity. These findings will be of help in providing useful information for future therapeutic studies of DU patients.

## Figures and Tables

**Table 1.** Causes of Neurogenic Bladder in 32 Patients

Disease	DO-Related Voiding (20 Pts)	Voluntary Voiding (12 Pts)
Brain tumor	3	1
Epilepsy	1	0
Myelitis, spinal cord tumor	3	2
Parkinson’s disease	0	1
Multiple sclerosis	3	0
Pelvic surgery	1	3
Unknown	9	5

DO, detrusor overactivity.

**Table 2.** Assessment of Lower Urinary Tract Function Parameters in Patients with Detrusor-Overactivity-Related Voiding and Detrusor Overactivity with Voluntary Voiding

	DO-Related Voiding	Voluntary Voiding	*P*
No. pts.	20	12	
Qmax (mL/s)	8.9 ± 4.1	15.2 ± 5.0	<.01
Pdet.Qmax (cmH_2_O)	45.5 ± 22.3	25.9 ± 12.7	.01

DO, detrusor overactivity; Pdet.Qmax, detrusor pressure at Qmax/maximum flow rate.
